# NMDARs Containing NR2B Subunit Do Not Contribute to the LTP Form of Hippocampal Plasticity: In Vivo Pharmacological Evidence in Rats

**DOI:** 10.3390/ijms22168672

**Published:** 2021-08-12

**Authors:** Abdallah Ahnaou, Kobe Heleven, Ria Biermans, Nikolay V. Manyakov, Wilhelmus H. Drinkenburg

**Affiliations:** Department of Neuroscience, Data Science, Janssen Research & Development, Division of Janssen Pharmaceutica NV, Turnhoutseweg 30, B-2340 Beerse, Belgium; kheleven@its.jnj.com (K.H.); rbierman@its.jnj.com (R.B.); nmanyak1@its.jnj.com (N.V.M.); wdrinken@its.jnj.com (W.H.D.)

**Keywords:** synaptic plasticity, hippocampus, LTP, NMDA receptor, ketamine, NR2B, neurodegenerative and psychiatric disorders

## Abstract

Synaptic plasticity is the key to synaptic health, and aberrant synaptic plasticity, which in turn impairs the functioning of large-scale brain networks, has been associated with neurodegenerative and psychiatric disorders. The best known and most studied form of activity-dependent synaptic plasticity remains long-term potentiation (LTP), which is controlled by glutamatergic N-methyl-d-aspartate) receptors (NMDAR) and considered to be a mechanism crucial for cellular learning and memory. Over the past two decades, discrepancies have arisen in the literature regarding the contribution of NMDAR subunit assemblies in the direction of NMDAR-dependent synaptic plasticity. Here, the nonspecific NMDAR antagonist ketamine (5 and 10 mg/kg), and the selective NR2B antagonists CP-101606 and Ro 25-6981 (6 and 10 mg/kg), were administered intraperitoneally in Sprague Dawley rats to disentangle the contribution of NR2B subunit in the LTP induced at the Schaffer Collateral-CA1 synapse using the theta burst stimulation protocol (TBS). Ketamine reduced, while CP-101606 and Ro 25-6981 did not alter the LTP response. The administration of CP-101606 before TBS did not influence the effects of ketamine when administered half an hour after tetanization, suggesting a limited contribution of the NR2B subunit in the action of ketamine. This work confirms the role of NMDAR in the LTP form of synaptic plasticity, whereas specific blockade of the NR2B subunit was not sufficient to modify hippocampal LTP. Pharmacokinetics at the doses used may have contributed to the lack of effects with specific antagonists. The findings refute the role of the NR2B subunit in the plasticity mechanism of ketamine in the model.

## 1. Introduction

Extensive evidence has shown a possible link between abnormalities in glutamate neurotransmission and synaptic plasticity in neurodegenerative and psychiatric disorders [1,2,3,4,5,6,7]. Glutamate receptors mainly consist of N-methyl-D-aspartic-acid (NMDA) and α-amino-3-hydroxy-5-methylisoxazole-4-propionic acid (AMPA) receptors. The kinetic and biophysical properties of NMDARs and AMPARs are mainly determined by the composition of their corresponding subunit [4,8,9]. The NMDA receptor acts as an activity-dependent coincidence detector in the central nervous system (CNS), which is commonly identified with the induction of two forms of synaptic plasticity, long-term potentiation (LTP) and long-term depression (LTD), the dominant experimental models of synaptic plasticity and learning [5,6,10,11,12,13]. A major feature of LTP is the requirement of activation of NMDA receptors, which are heteromultimers assemblies comprising two obligatory GluN (also termed NR) 1 subunits and two modulatory subunits, either NR2 or NR3 [14]. There are four NR2 subunits (A–D) in the brain [15], while NR2A, NR2B and some NR1 are the central NMDAR subunits expressed in the brain of rodents. These majorities of NMDAR subunits mainly ensure the association of the NR1–NR2A and NR1–NR2B complexes as heteromeric NMDARs at the synaptic level [4,16,17,18]. The NR2B and NR2A subunits are structurally and functionally distinct, providing properties unique to NMDAR function in transmission and basal synaptic plasticity. In addition, these two NMDAR subunits are expressed at different times of development: GluN2B is predominant in early postnatal development [19], while GluN2A levels gradually increase during development and ultimately exceed those of GluN2B [20,21,22]. NMDARs containing NR2B preferentially target extrasynaptic sites, while NMDARs containing NR2A are localized at the postsynaptic density [23].

Synaptic plasticity is the biological process by which specific patterns of synaptic activity cause changes in synaptic strength and are believed to contribute to learning and memory [24]. NMDAR antagonists may disrupt memory processes through blockade of NMDAR-dependent synaptic plasticity including the formation and maintenance of LTP. Ketamine, a pan-NMDAR channel blocker, elicits several symptoms of schizophrenia in healthy subjects, and disrupts normal behavior and cognitive function in humans and experimental animals [25,26,27,28,29,30], as well as eliciting favorable antidepressant effects that last up to a week with minimal adverse side effects [31,32,33]. Due to its rapid onset and short half-life, the lasting effects are likely mediated by changes in synaptic-related proteins, synaptic plasticity and/or synaptic plasticity deriving from modulation of different NMDAR subunits.

The heterogeneity of the distribution of NMDAR subunits in the CNS has encouraged the development of subtype-selective compounds with the possibility that these compounds may not have the behavioral side effects seen with non-selective NMDAR antagonists. Evidence indicates that ketamine’s effect likely results from its antagonism of NR2B-subunit-containing NMDAR. Since ketamine equally blocks NR2A- and NR2B-containing NMDAR, and has affinity to other receptors, drugs selective for NR2B may have improved therapeutic efficacy and side effect profile. Therefore, selective NR2B subunit antagonists have been developed, including CP101606 and Ro 25-6981 [34]. However, divergences in the literature have emerged over the past two decades as to the role of these different NMDAR subunits in the LTP and LTD forms of synaptic plasticity [15,35,36], see also Table 1.

The high number of in vitro studies disagrees with the limited studies of in vivo plasticity after pharmacological modulation, which therefore limits their translatability. Understanding the contribution of NMDAR subunits in synaptic plasticity deficits, which is a current hypothesis in neurodegenerative and psychiatric diseases, can help design better drugs without adverse cognitive effects. This study aimed to compare the effects of ketamine and two different types of selective NR2B antagonists. In a single-dose pharmacological design, the NMDAR antagonist ketamine (5 and 10 mg/kg) and the selective NR2B antagonists CP-101606 [43,44] (6 and 10 mg/kg) and Ro 25-6981 (6 and 10 mg/kg) were used to explore the contribution of the NR2B subunit in the vivo LTP response. NR2B antagonists bind to the interface of the NR2B/NR1 amino terminal domains to allosterically reduce the probability of channel opening to inhibit ion flux and functionally inhibit receptor activity [45,46]. In the combined pharmacological design, ketamine and CP-101606 were administered consecutively to examine the relevance of the NR2B subunit in the mediating effect of ketamine on LTP response.

## 2. Results

### 2.1. Input/Output (I/O) Criteria

**Figure 1:**
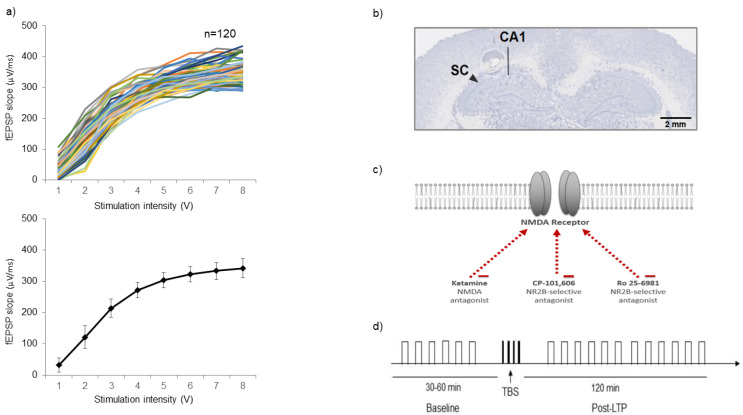
(**a**) Spaghetti plot of individual input/output curves and average values. Historical individual fEPSP slopes (*n* = 120) lie between 280 and 430 µV/ms at 200 µs stimulus duration and the calculated stimulus fit between 1.75 and 3.25 V. The average calculated fEPSP slope is 341.97 ± 31.24 µV/ms. (**b**) Stained and scanned slide for verification of the stimulating and recording electrode’s positioning (stimulation electrode at the ipsilateral SC pathway, recording electrode at the CA1 region of the hippocampus, more specifically in the stratum radiatum layer). (**c**) Pharmacological compounds used to study LTP response in SD rats, including ketamine, CP-101606 and Ro 25-6981. (**d**) Schematic presentation of the TBS electrical stimulation protocol that induce LTP at the SC-CA1 synapses in anesthetized rats.

I/O curves that were generated before the LTP induction protocol were used as the first gate to establish inclusion and exclusion criteria. Stimulation at intensities ranging from 1 to 10 V in steps of 1 V at 0.033 Hz frequency and 200 µs duration were delivered and three responses were recorded at each intensity. Individual fEPSP slope followed a sigmoid curve distribution and the calculated test stimulus fit between 3 and 4 V for all experiments (Figure 1a). A representative of the site of electrical stimulation and recording verified with standard histology technique is shown in Figure 1b. The NMDAR antagonist ketamine, and the specific NMDAR containing NR2B subunit antagonists CP-101606 and Ro 25-6981, were used to pharmacologically modulate LTP response to TBS tetanization protocol (Figure 1c,d).

### 2.2. Effects of Ketamine on 5xTBS LTP Response

**Figure 2:**
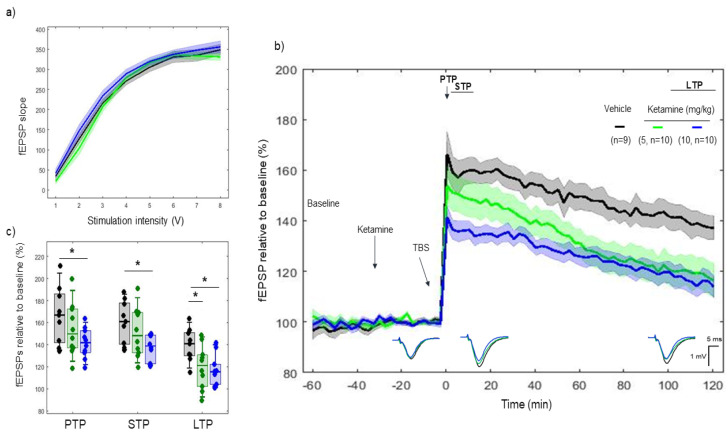
LTP response to ketamine (5 and 10 mg/kg). Ketamine consistently reduced the TBS-induced LTP response for both doses of 5 and 10 mg/kg. (**a**) Collective input/output curves of stimulation voltage and fEPSP slope overlap and no difference is observed in basal synaptic excitability prior treatment with ketamine. (**b**) Basal fEPSPs before tetanization were considered indifferent while after tetanization, the LTP responses of ketamine-treated animals for both doses (green, *n* = 10; blue *n* = 10) were lower than the LTP responses in the control group (black, *n* = 9). Values represent mean ±95% confidence intervals. Outsets under the LTP curves represent average waveform field potentials during 30 min baseline prior to tetanization, 0–30 min after tetanization and 90–120 min after tetanization. Horizontal bar: 1 mV, vertical bar: 5 ms. (**c**) PTP, STP and LTP responses boxplots, * *p* < 0.05.

Ketamine, an NMDA antagonist, was administered 30 min before tetanization. I/O curves showed no difference in basal synaptic excitability before tetanization (Figure 2a). A difference was found between vehicle and ketamine (10 mg/kg) on post-tetanic potentiation (PTP) (*p* = 0.016) (166.47 ± 16.9% vs. 141.47 ± 8.50%, respectively), but not between vehicle and ketamine (5 mg/kg) (*p* = 0.28) (166.47 ± 16.9%, vs. 153.84 ± 14.80%, respectively). Likewise, ketamine (10 mg/kg) reduced short-term potentiation (STP) (*p* = 0.006) (160.43 ± 12.7% vs. 137.46 ± 7.40%, respectively), whereas no such effect was observed with ketamine (5 mg/kg) (*p* = 0.35) (160.43 ± 12.7% vs. 151.18 ± 14.04%, respectively). Furthermore, a difference was found between vehicle and both 5 and 10 mg/kg on LTP (*p* = 0.02) (139.65 ± 9.78% vs. 119.58 ± 12.17%), and (*p* = 0.003) vs. 116.84 ± 8.61%, respectively. (Figure 2b,c)

When comparing the rate of degradation over the 2 h, the linear mixed effect model revealed a consistent effect of ketamine (5 mg/kg) on LTP degradation after the TBS tetanization vs. vehicle (model’s slope: (*p* < 0.001)), but not with ketamine (10 mg/kg) (model’s slope: (*p* = 0.42)).

### 2.3. Effects of CP-101606 on 5xTBS LTP Response

**Figure 3:**
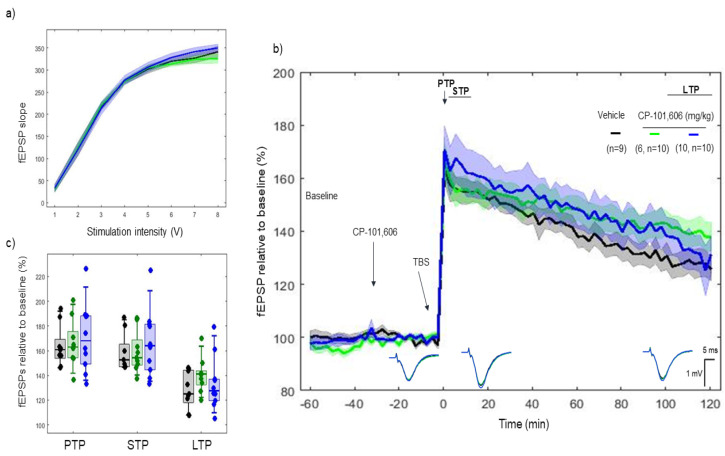
LTP response to CP-101606 (6 and 10 mg/kg). CP-101606 did not reduce the TBS-induced LTP response for both doses of 6 and 10 mg/kg. (**a**) Collective input/output curves of stimulation voltage and fEPSP slope overlap and no difference is observed in basal synaptic excitability prior to treatment with CP-101,606. (**b**) Basal fEPSPs before tetanization were considered indifferent as well as after tetanization, and the LTP responses of CP-101606-treated animals for both doses (green, *n* = 10; blue, *n* = 10) were not different from the LTP responses in the control group (black, *n* = 9). Values represent mean ±95% confidence intervals. Outsets under the LTP curves represent average waveform field potentials during 30 min baseline prior to tetanization, 0–30 min after tetanization and 90–120 min after tetanization. Horizontal bar: 1 mV, vertical bar: 5 ms. (**c**) PTP, STP and LTP responses boxplots, *p* < 0.05.

CP-101606, a selective NR2B antagonist of the NMDA receptor, was administered 30 min before tetanization. I/O curves showed no difference in basal synaptic excitability before tetanization. (Figure 3a). No difference was found between vehicle, 6 mg/kg and 10 mg/kg on PTP (*p* > 0.05) (163.64 ± 10.8%, 166.24 ± 12.62% and 170.77 ± 17.35%, respectively), STP (*p* > 0.05) (158.06 ± 9.94%, 159.14 ± 10.99% and 166.54 ± 17.01%, respectively), and LTP (*p* > 0.05) (128.19 ± 9.68%, 140.25 ± 9.22% and 133.50 ± 13.55%, respectively) (Figure 3b,c).

LTP responses throughout the 2 h course after TBS degraded slower with 6 mg/kg (model’s slope: vehicle vs. CP-101606 (6 mg/kg) (*p* < 0.001)), whereas no such effect was found in the speed of LTP degradation with CP-101606 10 mg/kg (model’s slope: (*p* = 0.75).

### 2.4. Effects of Ro 25-6981 on 5xTBS LTP Response

**Figure 4:**
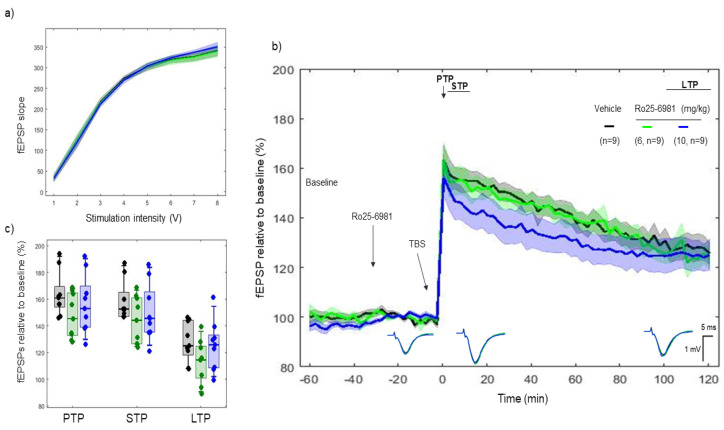
LTP response to Ro 25-6981 (6 and 10 mg/kg). Ro 25-6981 did not alter the TBS-induced LTP response for both doses of 6 mg/kg and 10 mg/kg. (**a**) Collective input/output curves of stimulation voltage and fEPSP slope overlap and no difference is observed in basal synaptic excitability prior treatment with Ro 25-6981. (**b**) Basal fEPSPs before tetanization were considered indifferent as well as after tetanization, the LTP responses of Ro 25-6981 treated animals for both doses (green, *n* = 9; blue, *n* = 9) were not different from the LTP responses in the control group (black, *n* = 9). Values represent mean ± 95% confidence intervals. Outsets under the LTP curves represent average waveform field potentials during 30 min baseline prior to tetanization, 0–30 min after tetanization and 90–120 min after tetanization. Horizontal bar: 1 mV, vertical bar: 5 ms. (**c**) PTP, STP and LTP responses boxplots, *p* < 0.05.

Ro 25-6981, another selective NR2B-antagonist of the NMDAR, was administered 30 min before tetanization. I/O curves showed no difference in basal synaptic excitability before tetanization. (Figure 4a). No difference was found between vehicle, 6 mg/kg, and 10 mg/kg on PTP (*p* > 0.05) (163.64 ± 10.82%, 163.6 ± 12.67%, and 156.19 ± 14.40%, respectively), STP (*p* > 0.05) (158.06 ± 9.94%, 156.98 ± 11.35%, and 150.8 ± 13.95%, respectively) and LTP (*p* > 0.05) (128.19 ± 9.68%, 125.82 ± 8.60%, and 125.06 ± 12.27%, respectively) (Figure 4b,c).

LTP responses through the 2 h course after TBS degrade faster with Ro 25-6981 (10 mg/kg) (model’s slope: vehicle vs. Ro 25-6981 (10 mg/kg) (*p* < 0.001)), whereas no such effect was found in the speed of LTP degradation with Ro 25-6981 (6 mg/kg) (model’s slope: vehicle vs. Ro 25-6981 (6 mg/kg) (*p* > 0.05)).

### 2.5. Effects of Combined Pharmacology of CP-101606 and Ketamine on 5xTBS LTP Response

**Figure 5:**
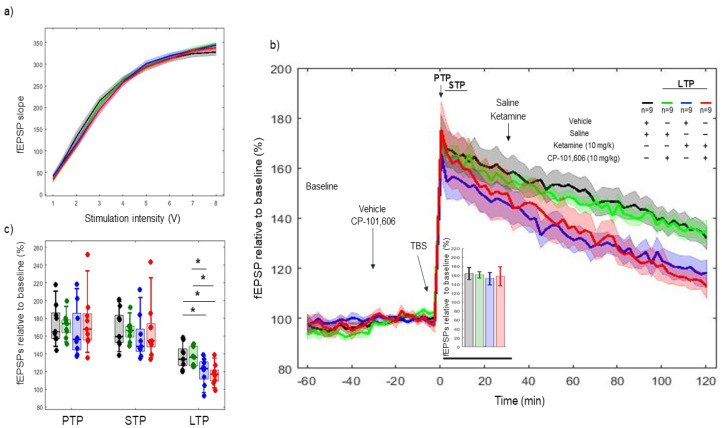
LTP response to a combined pharmacology of CP-101606 (10 mg/kg) and ketamine (10 mg/kg) reduced the TBS-induced LTP response. (**a**) Collective input/output curves of stimulation voltage and fEPSP slope overlap and no difference was observed in basal synaptic excitability prior to treatment with CP-101606 (vehicle) and ketamine (saline). (**b**) Basal fEPSPs before tetanization were considered indifferent while after tetanization, the LTP responses of CP-101,606–ketamine (red, *n* = 9) and vehicle–ketamine treated animals (blue, *n* = 9) were lower than the LTP responses in the CP-101,606–saline (green, *n* = 9) and vehicle–saline (black *n* = 9) groups. Values represent mean ± 95% confidence intervals. (**c**) PTP, STP and LTP responses boxplots, * *p* < 0.05.

CP-101606 and ketamine, a selective NR2B antagonist of NMDAR and an NMDA antagonist, respectively, were used. CP-101606 was administered 30 min before tetanization and ketamine 30 min after tetanization. I/O curves showed no difference in basal synaptic excitability before tetanization (Figure 5a). No difference was found between vehicle–saline, vehicle–ketamine (10 mg/kg) and CP-101606 (10 mg/kg)–ketamine (10 mg/kg) combinations on PTP (*p* = 0.52 AND *p* = 0.88) (173.51 ± 14.84%, 165.5 ± 18.70% and 175.54 ± 21.69%, respectively) and STP (*p* = 0.37 and *p* = 0.88) (168.18 ± 13.80%, 158.14 ± 16.33% and 166.66 ± 21.55%, respectively). However, a consistent effect was found between vehicle–saline, vehicle–ketamine (10 mg/kg) and CP-101606 (10 mg/kg)–ketamine (10 mg/kg) combination on LTP (*p* = 0.023, *p* = 0.004) (137.12 ± 8.89%, 120.30 ± 9.59% and 116.98 ± 7.54%, respectively) (Figure 5b,c). Likewise, significant differences (*p* = 0.023) were found between CP-101606 (10 mg/kg)–saline and CP-101606 (10 mg/kg)–ketamine (10 mg/kg) groups. Note that no difference was found in fEPSP between groups during the first 30 min after tetanization (Figure 5b, bar plot).

LTP responses throughout the 2 h course after TBS degraded faster with vehicle–ketamine (10 mg/kg) and CP-101606 (10 mg/kg)–ketamine (10 mg/kg) vs. CP-101606 (10 mg/kg)–saline (model’s slope: (*p* < 0.001) and (*p* < 0.001), respectively), using a mixed effect model.

## 3. Discussion

The current results provide further evidence that ketamine suppresses the LTP response at the doses tested. The selective NR2B antagonists CP-101606 and Ro25-6981 were equally ineffective in causing changes in the LTP response. Administration of CP-101606 before TBS did not influence the effects of ketamine when administered half an hour after tetanization, suggesting a limited contribution of the NR2B subunit in the in the mechanisms of ketamine action.

Before the start of the LTP induction protocol, the I/O curves were used as the first inclusion and exclusion criteria. The collective I/O curves between stimulation voltage and fEPSP slope overlap with historical data, showing broadly similar basal synaptic excitability. The subsequent changes observed in fESPSs can be attributed to the pharmacological treatment administered during the LTP experiment.

The TBS induction LTP protocol has generated interest in recent years because it is believed to approximate a relevant physiological excitability model and mimic the EEG activity of the endogenous theta frequency (4–8 Hz) recorded in the hippocampus when the animal is engaged in learning and memory functions. TBS is a repeating pattern of short bursts of pulses (e.g., four pulses at 100 Hz) with brief pauses (∼200 ms) between bursts [18,47,48,49]. Long-lasting plasticity is expressed sequentially in different phases over time. The application of brief stimuli to the SC synapse consists of three different phases, including PTP, STP and LTP. First, PTP is of presynaptic origin, which has a short onset and lasts between 30 s and a few minutes [50]. Here, the presynaptic accumulation of calcium causes PTP. Subsequently, the effect of PTP is readily diminished by the clearance of calcium. In this phase, PTP is independent of NMDAR. Unlike PTP, the subsequent STP and LTP phases are of postsynaptic origin and are therefore forms of potentiation dependent on NMDAR [24,51,52,53,54,55].

### 3.1. Ketamine, a Low-Affinity Antagonist of the NMDA Receptor, Decreased the LTP Response

Doses of ketamine were based on previous behavioral effects in animal models of depression and experimental pharmacokinetic modeling studies in rats, which showed optimal exposure levels closely correlated with exposure levels of the antidepressant therapeutic dose in clinical trials [56,57]. Ketamine can interact with any composition of NMDAR subunits [58,59]. Ketamine can interact with di-heteromeric (e.g., NR1–NR2A and NR1–NR2B) and tri-heteromeric (e.g., NR1–NR2A–NR2B) receptors, constituting two or three different subunits, respectively. Despite the relatively lower affinity of ketamine, having the full spectrum of NMDAR as a target could be a reason why a minimum dose of 5 mg/kg was already able to block enough NMDAR and induce a change in the LTP response. Although the effect of ketamine is known to be mediated through NMDAR-mediated excitatory postsynaptic currents (EPSCs) recorded from CA1 pyramidal cells, the reduced LTP response can also be achieved via other systems, such as dopaminergic, adrenergic and monoaminergic systems as well as non-NMDA glutamatergic systems via AMPA receptors [58,60], which can cause undesirable effects such as dissociative and psychotomimetic effects [61,62]. Thus, the risk/benefit ratio limits the therapeutic potential of ketamine for CNS diseases, and the heterogeneity of the distribution of NMDAR subtypes in the CNS has encouraged the development of subtype-selective compounds with the possibility that these compounds may not have the behavioral side effects seen with non-selective NMDAR antagonists.

### 3.2. CP-101606 and Ro 25-6981, High-Affinity and Selective NR2B Antagonists of the NR2B Subunit, Did Not Modify the LTP Response

CP-101606, known as traxoprodil, and Ro 25-6981 are highly selective NR2B subunit antagonists with no affinity for the NR2A subunit [34,63,64]. In auditory evoked potentials in rats, CP-101606 robustly and dose-dependently inhibited deviant’s N1 amplitude, and consequently abolished the pre-attentive deviance detection [65], whereas CP-101606 increased the amplitude of auditory gating after drug elimination [28]. Quantitative electroencephalography in cynomolgus monkeys, showed that CP-101606 elicited robust decrease in the beta frequency band [66]. Here, no major changes were observed in the vivo LTP response, which could be explained by several potential reasons, including the unbalanced composition of NMDAR subunits. Both di-heteromeric and tri-heteromeric receptors are expressed in the brain of rodents. Among all the multimer receptor complexes, NR2A and NR2B subunits are mainly di-heteromeric receptors [67]. Regarding the evolution of the NR2A/NR2B ratio mentioned above as rodents age, younger ages are more associated with lower ratios and therefore are predominant in NR1–NR2B complexes [68]. In contrast, developed and older rats contain more NR1–NR2A complexes, resulting in a higher NR2A/NR2B ratio [69]. Since CP-101606 does not inhibit NR2A di-heteromeric complexes, a weak to no response after selective modulation of the NR2B subunit by CP-101606 could be due to decreased abundance and expression of the NR2B subunit in these NMDA di-heteromeric receptors by age [70].

Other studies highlight the role of NR2B as one of the most associated subunits in the tri-heteromeric receptor family [71]. Tri-heteromeric receptors are the most abundant NMDAR complexes in rodent brains, while di-heteromeric receptors containing NR2B represent only less than a third [70]. Therefore, the effect on the LTP response via NR2B subunit in the tri-heteromeric complex may possibly counterbalance the relatively smaller blockade of NR2B via di-heteromeric receptors [70]. Remarkably, CP-101606 binds with high affinity to the relatively less abundant di-heteromeric receptor family compared to the tri-heteromeric receptor family, whereas Ro 25-6981 has been assessed to have a high affinity for tri-heteromeric receptors [63]. It is believed that the difference in affinity properties depends on the other subunits integrated into these tri-heteromeric receptors. The difference between NR1–NR2B di-heteromeric receptors and NR1–NR2A–NR2B tri-heteromeric receptors is the integration of an additional NR2A subunit. This subunit is believed to cause the lower affinity of CP-101606 to bind to tri-heteromeric receptor complexes, while it does not change the high-affinity properties of Ro 25-6981 for the sub-NR2B unit integrated in a tri-heteromeric receptor. Therefore, CP-101606-induced occupation of 80% NR2B only reached a maximum inhibition of 30% within these tri-heteromeric NR1–NR2A–NR2B receptors [70]. Unlike the low selectivity of ketamine, CP-101606 only works very selectively through the NR2B receptor. This mechanism of action may therefore not cause an alteration of the LTP response due to the weak inhibition of the predominant and major tri-heteromeric receptor complexes.

Other factors such as stimulation protocols may be responsible for the lack of effect after administration of CP-101606. While TBS stimulation will only use NMDA receptors, high-frequency stimulation uses both NMDA receptors and voltage-gated calcium channels as the underlying mechanism of action to induce LTP. Due to their different underlying mechanisms, divergent conclusions can be drawn. Although no clear association was often observed between NR2B and LTP after stimulation with TBS, high-frequency stimulation frequently linked NR2B and LTP (Table 1). Thus, the stimulation protocol used can influence the results and subsequent conclusions about the link between NMDA subunits and synaptic plasticity of LTP.

In addition, in vitro tests revealed that the LTP response after modulation by CP-101606 was reduced in NR1–NR2B receptor complexes, whereas on the contrary, the LTP response was not affected in NR1–receptor complexes with NR2A. In addition, no altered LTP response was observed after administration of lower doses of Ro 25-6981 [9], whereas higher doses markedly decreased the LTP response [40]. Along with the subunit composition hypothesis, in which a relatively higher abundance of tri-heteromeric receptors compared to di-heteromeric receptors has been established in rodent brains, a possible change may occur. As an in vitro environment is more controlled and often less predictive than in vivo models, more isolated results can be obtained. Thus, it is possible that CP-101606 effectively inhibits the NR1–NR2B complexes, but that these are still only a minority compared to the predominant tri-heteromeric complexes and that the overall alterations of the LTP response remains weak.

Moreover, the pharmacokinetics of NR2B antagonists could be another variable to explain these differences in LTP responses, as well as the limited translatability of in vitro results to in vivo results [72]. Since these in vitro tests are performed on sections of the hippocampus, potential pharmacokinetic effects in the animal model are minimized. The effect in vitro and the minimal effect in vivo could be explained by certain limitations of the pharmacokinetics of the compounds used. Limited absorption after intraperitoneal injection, insufficient distribution once absorbed into the blood or extensive excretion mechanisms can result in short exposure levels in the blood and body reaching the site of action in the hippocampus. Specifically, other studies have reported extensive metabolism and rapid excretion within 48 h of administration. Nevertheless, dosing and quantification occurs within two hours and should not be altered by extensive metabolism or elimination. However, the levels at the site of action after crossing the blood–brain barrier, the activity of the metabolites formed, and the time of action could be questioned. Thus, the limited exposure levels and the time spent at the site of action could explain the difference between the altered results in vitro and lesser effect of CP-101606 and Ro 25-6981 on the LTP response in vivo compared to effects observed in vitro [11,73,74,75].

Furthermore, a divergent metabolism was also noted after administration of a single dose of this compound (50, 100 or 300 mg intravenously by infusion), depending on the genetic polymorphism of the animal. Whereas CYP2D6 poor metabolizers followed a more linear and dose-independent clearance, CYP2D6 rapid metabolizers followed non-linear dose-dependent kinetics. In addition, regarding oral bioavailability, distinct results were observed, of 80% and 20 to 60%, respectively, for poor metabolizers and extensive metabolizers. The nonlinear kinetics of extensive metabolizers is probably due to the saturation of the CYP2D6 enzyme itself. Unlike the in vivo LTP study where normal SD rats were used, a possible genetic polymorphism, regarding the metabolism of animal models of CNS disorders, should be investigated to correctly interpret the corresponding LTP results [76,77].

The route of administration may also support these pharmacokinetic considerations. A difference in effect on the LTP response when using two separate delivery methods was found [42]. When Ro 25-6981 was injected intraperitoneally, no effect on the LTP response was observed. In contrast, when Ro 25-6981 was administered by intrahippocampal injection, a reduction in the LTP response was observed. This argument asserts that the route of administration, and therefore the pharmacokinetics in the animal model, could mask any effect of a selective NR2B antagonist agent on the LTP response as an explanation for the differences between in vitro and in vivo results.

### 3.3. In Combined Pharmacology, CP-101606 Did Not Influence the Effects of Ketamine

In the combined pharmacology study, we investigated whether modulation of NR2B activity could interfere with the effect caused by ketamine. While CP-101606 saline did not alter the LTP response, vehicle–ketamine and CP-101606–ketamine decreased the LTP response 120 min after tetanization. The vehicle–ketamine EPSP and CP-101606–ketamine curves follow a similar decrease suggesting a limited contribution of the NR2B subunit in the effect of ketamine on LTP.

While the mechanism of action of CP-101606 via the NR2B subunit has been described, the mechanism of action of ketamine is not yet entirely clear [62]. Since the LTP response to ketamine did not change with or without prior administration of CP-101606, the compounds likely mediate their effects via distinct mechanisms. If ketamine would also have interacted with the NR2B subunit, the decreasing effect of CP-101606–ketamine on the LTP response should be weaker than vehicle–ketamine since CP-101606 would have already (partially) bound the binding sites of the NR2B subunit. However, depending on the reversibility of the binding of CP-101606 to the NR2B subunit, ketamine could compete for the binding sites and previously bound CP-101606 could be released, resulting in the same decrease in the LTP response alike the altered LTP response observed with vehicle–ketamine. The competition and the corresponding relative occupancy levels depend on the doses of the two compounds used, the concentration at the site of action and a high affinity towards other targets (mainly for ketamine). However, these scenarios have been overlooked, since ketamine has a lower affinity than CP-101606 for the NR2B subunit of NMDAR; the two compounds were assayed equally and were equally potent [64].

In particular, the revelation of the distinct mechanisms of action of ketamine and CP-101606, based on the observed difference in the LTP response, should be carried out with caution. For the establishment of single and combined dose pharmacology, an assumption was made to interpret the results of these study designs. The hypothesis included that the NR2B subunit is present and sufficiently abundant in the brain region of the recording site, specifically the CA1 region of the hippocampus [21,78]. However, since the composition of the subunits dynamically differs between different regions of the brain such as the hippocampus but also the striatum and cortex, this assumption should be applied with caution.

Ketamine does not affect all NMDARs but rather affects the NR2A and NR2B subunits. CP-101606 and Ro 25-6981, the two selective NR2B antagonists, did not alter the LTP response. A predominance of the NR2A subunit in the hippocampus and cortex in contrast to the predominance of the NR2B subunit in other regions such as the striatum could be a counterargument to the lack of effect on LTP [79,80,81]. In addition, within the combined pharmacology, the conclusion that ketamine and CP-101606 act via different mechanisms of action should also be interpreted with caution. Thus, in the overall functional context of the channel, the NR2A rather than NR2B subunit may shorten the channel open time and affect receptor kinetics, with a potential impact on long-term plasticity.

### 3.4. Perspective

Due to their therapeutic relevance in recent decades, ketamine and CP-101606 have been studied for their exposure levels of high clinical efficacy with few side effects. Ketamine was already recognized as a potential therapy in several types of depressive disorders, while the exact mechanism of action remained uncertain [61]. The reasons for the beneficial clinical results are probably the rapid and long-lasting changes in plasticity processes. Ketamine was thought to have clinically relevant effects via the NR2B subunit for the treatment of patients with treatment-refractory major depressive illness. These effective results, which contrast with the lack of effect in the acute in vivo LTP study, raise important questions about the long-lasting changes in synaptic efficacy after NR2B blockade [82]. Attempts have been made to understand the pharmacokinetics of this compound to provide additional bases for translation across different species.

One study used a wide range of subcutaneous doses of ketamine (1 to 100 mg/kg) and standardized these doses via plasma concentration unrelated to the level of NMDAR occupancy that each dose induces in different species, including rodents, non-human primates and humans. The study provided a decent basis to compare interspecific receptor occupancy and the associated observed pharmacodynamics induced by modulation of NMDAR [57]. Future in vivo LTP studies using intrahippocampal versus intravenous routes of administration as well as different dosing regimens of NMDAR occupation could help to further elucidate the divergent results.

In addition, research has been carried out on possible pharmacokinetic differences in a healthy or pathological condition. One study reported a different metabolism after administration of ketamine (0.5 mg/kg, intravenous) depending on the diagnosis previously made. Patients with major depressive disorder had different metabolite profiles than patients with bipolar depression. This is important because both the active compound and the metabolites may or may not be responsible for side effects. While in this in vivo LTP study no animal model of CNS diseases was used, further elaboration on possible altering metabolite profiles in corresponding disease animal models is needed to correctly interpret the corresponding LTP results.

Another study examined the functional effects of ketamine and CP-101606, via recording of the local field and auditory evoked potentials. While in the LTP in vivo study, only short-term effects were observed within two hours post tetanization, this study reported distinct effects before (5 to 30 min drug on period) and after complete drug elimination (5 to 6 h drug off period). While ketamine and CP-101606 both increased the auditory evoked response after drug elimination, only ketamine had an acute effect during the drug on period [28]. These findings suggest possible different mechanisms of action between these two compounds and raise the question of designing more selective drugs that maintain the desirable acute effects of ketamine, illustrated in this study, while avoiding possible correlated side effects such as psychotomimetic symptoms.

Memories formed in adulthood can be memorized for years, while similar memories formed in childhood seem to be easily and quickly forgotten. Infantile or childhood amnesia is the inability of adult humans to recall episodic experiences that occurred in the first few years of life (typically 0 to 3 years of age) and the tendency to have sparse memories of episodic experiences that occurred before the age of 10 [83,84,85,86]. Studies of infantile hippocampal learning mechanisms described developmental time windows during which the brain is particularly sensitive and receptive to experiences, and the findings of which may help shed light on the mechanism by which forgetting rates may vary with age [87,88,89]. NMDAR has been associated with cognitive [90] and the change in the development of NMDAR from predominantly NR2B to predominantly NR2A determines the sensitivity of enhanced network connections to depotentiation [91]. At the cellular level, memory formation involves a prolonged strengthening of neuronal connections via LTP, and long-term storage of information requires the maintenance of LTP. Depotentiation at specific sets of synapses erases memory, a mechanism by which learning new information can alter the stability of previously stored memory traces to produce forgetting, possibly by deconstructing engram cell connectivity [92,93,94]. Artificially increasing the NR2A/NR2B ratio by lentivirus-mediated NR2A overexpression or downregulation of NR2B in the mouse hippocampus promoted depotentiation without producing apparent deleterious effects on LTP. As a result, animals could learn the fear conditioning task normally, whereas only memory retention was particularly impaired, indicating a key role of the NR2A subunit component in memory loss [95]. Assuming the correlation between depotentiation and memory loss, in the present work, TBS-induced LTP remained stable after blockade of the NR2B subunit, indicating no role in depotentiation for this sub-type NMDAR. Therefore, NR2B modulators may be effective without causing the deficits in hippocampus-dependent learning and memory characteristic of nonselective NMDAR antagonists. As the age dependence of depotentiation is controlled by the ratio of NR2A/N2B subunits, future studies should explore whether antagonism of NMDARs containing GluN2B in adolescent animals mimicking a decrease in the GluN2A/GluN2B ratio would cause depotentiation.

Other studies recently suggested the use of CP-101606 to reduce dyskinetic symptoms induced by L-Dopa. Two study designs were performed, consisting of high- and low-dose groups (2.22 and 0.74 mg/kg). Although dyskinetic symptoms improved in a dose-dependent manner, cognitive impairment was considered more of a side effect in the high-dose group. Compared to the in vivo LTP results, this study demonstrated the important balance between the possible effect and the side effects of CP-101606 and required an adequate therapeutic balance concerning the benefit/risk ratio [96].

To sum up, the previous hypotheses, such as the dynamic composition of the subunits, the expression in different regions of the brain, the different underlying mechanisms due to the method of stimulation chosen and the distinct pharmacokinetic properties of the compounds used, have attempted to shed light on the link between NMDA receptors and synaptic plasticity. Nonetheless, there is still a long way to go, including exploration of the relevant research questions listed above, before realizing a potential breakthrough of NMDAR subunits in the treatment of CNS disorders.

### 3.5. Conclusion

Discrepancies have been found in the literature regarding the specific role of the NMDA, NR2A and NR2B subunits in the modulation of synaptic plasticity. Laboratory parameters, distinct receptor subunit assemblies and their expression in different regions of the brain as well as the pharmacokinetic profile of the specific antagonists may explain divergent results between in vitro and in vivo. Ketamine, a non-selective NMDA antagonist, altered the LTP response, whereas CP-101606 and Ro 25-6981, the two selective NR2B antagonists, did not influence the magnitude of LTP. It is possible that pharmacokinetics at the doses used may have contributed to the lack of effects with specific antagonists. Consecutive administration of CP-101606 and ketamine reduced the LTP response, similar to effects of ketamine alone, suggesting the low contribution of the NR2B subunit in the mechanisms of ketamine action. Future studies involving intra-hippocampal injections of NR2B antagonists, testing of higher doses (e.g., 30 mg/kg) or targeting other regions of the brain (e.g., striatum, cortex) will hopefully allow us to ascribe specific functional roles of the NR2B subunit in synaptic plasticity. Additionally, NR2A-selective compounds should be designed and tested in the hippocampus as well as other regions of the brain to further clarify the role of NR2A subunits in synaptic plasticity. 

## 4. Materials and Methods

### 4.1. Animals and Surgical Procedures

The conduct of the experiments was strictly and fully in accordance with the international directives of the Association for Assessment and Accreditation of Laboratory Animal Care (AAALAC) and with the directive of the council of the European Communities of November 24, 1986 (86/609/EEC). The methods of these experiments were all approved by the local ethics committee. Male Sprague Dawley rats (SD, 180–395 g), acquired from Charles River Germany, were housed in individually ventilated cages, which were maintained in a controlled environment (sound attenuated, 22 +/−2c ambient temperature, relative humidity at 60% and a 12:12 light:dark cycle at a light intensity of 100 lux).

Before surgical procedures, the rats were treated with urethane (1.5 g/kg, intraperitoneal (i.p.) which could be supplemented by injections of 0.1–0.2 mg/kg (mg/kg). Paw pinch and tail pinch were used as indicators of the degree of anesthesia of the animal. If necessary, urethane was additionally supplemented (0.2 mL/100 g), to ensure complete anesthesia of the animal before the start of surgical procedures. A heating pad was used throughout the experiment to ensure that the rodent’s body temperature (37 °C) was maintained while a probe was used to monitor the constant temperature. After complete anesthesia, the rats were fixed in a motorized steretotactical frame (Stoelting, Wood Dale, IL, USA). A recording electrode (tungsten wire, 75 µm diameter, impedance 10 kΩ) was inserted into the stratum radiatum of the CA1 (CA1, AP −4.2 mm, mL −4.0 mm) and descended into the tissue 0.2 mm/min until a theta rhythm could be seen (DV: 2.5–3.4 mm). The bipolar stimulating electrode (tungsten twisted wires World Precision Instruments, 75 µm diameter, impedance 10 kΩ) was inserted into the Schaffer Collateral pathway (SC, AP −3.4 mm, ml 2.5 mm, DV: 1.9–2.4 mm).

### 4.2. Input/Output Functions

Before the full LTP experiment, the correct placement of SC-CA1 implants were finely adjusted by altering the depth of both stimulation and recording electrodes in 10 µm increments for optimal evoked field post-synaptic potentials (fEPSP) through the oscilloscope using single square pulses (200 µs, 100 µA). For stimulations, the settings of the stimuli were determined using a Labview homemade software, and stimuli were sent via a data acquisition board linked to a constant current isolator unit (Multichannel System MC STG4002). The evoked fEPSP responses of the SC fiber were passed through the active two-electrode Biosemi amplifier (differential amplifier, Netherlands). This response was digitized at a sampling rate of 3 kHz, and the data were saved to a file for offline analysis.

At the beginning of each experiment, an input/output (I/O) curve was generated (average of 3 pulses of 200 µs from 1–10 V in steps of 1 v at 0.033 Hz) to determine the voltages used during the LTP experiment.

### 4.3. In Vivo Electrophysiology LTP Protocol

Baseline synaptic transmission was measured without treatment for the period of (−60 until −30 min) and after treatments (−30 until 0 min). Post TBS tetanization, fEPSPs were recorded for the following 2 h using a previously described protocol [51,97], in which LTP response was induced with a 5 trains TBS protocol (see Table 2).

### 4.4. Histology

At the end of the electrophysiological study, three 30 s electrical pulses of 500 µA were produced which resulted in a lesion at the end tips of the stimulating and recording electrodes. The brains were harvested for histological verification of electrodes placement. Coronal sections of 20 µm thickness were sliced using a Leica CM 3050 cryostat-microtome (Leica, Belgium) and were examined using a light microscope. Animals with incorrect electrode placement were excluded from the study.

For staining, a standard counterstain method was applied. First, the slides were fixed in a solution of paraformaldehyde (4%, 4°C) for a period of 10 min. Next, the slides were washed twice, for a period of 5 min each, in a DAKO wash buffer. Thereafter, a solution of 0.5% Triton in PBS (x1) was used for a period of 10 min to permeabilize the cell membranes. Then, a second double washing step was set, for a period of 5 min each, in a DAKO wash buffer. Afterwards, they were placed in hematoxylin, 1/6 diluted in distilled water, for 2 min. The slides were then washed for 5 min under a stream of running tap water and then proceeded to dehydrate after this washing step. Dehydration occurred by immersion in a solution of 90% ethanol for 1 min, twice in 100% ethanol for 1 min each (in separate solutions) and then twice 1 min in xylene (in separate solutions). The slides were mounted using a permanent organic mounting medium (Vectamount^®^) onto 24 × 50 mm glass cover slides. Hereafter, the stained slides were investigated under a microscope (Zeiss Axioscan Z1 microscope) to find the precise position of the stimulation and recording electrodes. Finally, selected slides were scanned (Hamamatsu scanner) to create images of the stimulating and recording electrodes’ positions.

### 4.5. Drugs

Drugs were administered i.p. 30 min prior to tetanization in a volume of 1 mL/100 g body weight and compared to vehicle. Ketamine (5 and 10 mg/kg prepared in saline (NaCl), NR2B selective antagonists CP-101606 (6 and 10 mg/kg, formulated in CD10%–1NaCl–1 NaOH, intraperitoneal) and Ro25-6981 (6 and 10 mg/kg (in CD10%–1NaCl–1 NaOH), intraperitoneal) were also tested, characterized and validated in these animal models as potential antagonists. Lastly, a combined pharmacology was also performed. In this experimental design, selective NR2B antagonist CP-101606 (10 mg/kg) and NMDA antagonist ketamine (10 mg/kg) were combined and injected consecutively in time 30 min before and 30 min after tetanization, respectively.

### 4.6. Statistical Data Analysis

Data analysis was performed in the same manner as described previously [51,97]. The slope of the fEPSP was calculated using a least squares linear fit analysis on the 80% interval between the end of the artefact and the minimum of the negative peak. An fEPSP slope was recorded every 2.5 min, calculated by taking the average of five responses at 0.033 Hz. These fEPSP slopes were hereafter expressed as mean percentage change from the fEPSP during baseline period (baseline period during the last 30 min prior TBS tetanization). Group-averaged time course of fEPSP was formed together with 95% confidence intervals around the calculated mean.

The short-term potentiation (STP) value was defined as an averaged value in changes of fEPSP slopes (in comparison to baseline) for the time of 1–10 min after TBS. The LTP value was defined as an averaged value in fEPSP slope change (in comparison to baseline) for the period of 100–120 min after stimulation by TBS. The post-tetanic potentiation (PTP) was defined as an immediate fEPSP value of changes (in comparison to baseline) after TBS. PTP, STP and LTP results are presented as boxplots. Between groups, statistical comparison of PTP, STP and LTP was performed using analysis of variance (ANOVA) followed by a post hoc test (Dunnett’s test). * *p* < 0.05 was considered statistically significant. Results are reported as mean value ± sem.

To assess the speed of degradation after application of TBS, mixed-effect modeling was applied. When needed, time variable after TBS was log-transformed as t_new_ = ln(1 + *k* · t_old_), where t_old_ is real time expressed in minutes. This transformation allowed us to linearize the data. Coefficient *k* was adjusted for each data individually. Then, fEPSP relative to baseline (%) variable was modeled as t_new_ * group + (1|animal), where group variable is a categorical variable describing different conditions assessed during the test. Effect of group variable on a slope of the model (to assess differences in degradation of fEPSP responses over time) was tested. *p* < 0.05 was considered statistically significant.

## 5. Conclusions

This work demonstrated that ketamine suppressed the LTP response at the hippocampal SC-CA1 synapse, while blocking the NR2B subunit was not sufficient to modify LTP magnitude. Pharmacokinetics at the doses used may have contributed to the lack of effects with specific antagonists. The results provide further evidence for the role of NMDAR in the LTP form of synaptic plasticity in the rat hippocampus and refute the role of the NR2B subunit in the plasticity mechanism of ketamine in the model.

## Figures and Tables

**Table 1:** Example of disparate properties of plasticity synaptic forms associated with NMDAR containing NR2 subunits subtype.

Study Type	Stimulation Protocol	Synaptic Plasticity—NMDA Receptors Subtype Relation	Reference
In vitro slices Hippocampus, Sprague Dawley rats	Theta-burst stimulation for LTP	A correlation between the NR2A subunit and synaptic plasticity form LTP	[37]
Hippocampus, Sprague Dawley rats	Theta-burst stimulation for LTP	A correlation between the NR2A subunit and synaptic plasticity form LTP	[37]
Hippocampus, Lister Hooded rats	Low-frequency stimulation for LTD	The NR2A subunit is responsible for subsequent mechanisms of LTD.	[38]
Hippocampus, Long Evans rats	Low-frequency stimulation for LTD	The involvement of the NR2B subunit in the synaptic plasticity form LTD.	[39]
Hippocampus, Wistar rats	High-frequency stimulation for LTP and low-frequency stimulation for LTD	The regulation of LTP and LTD by both NR2A and NR2B subunits.	[15]
Hippocampus, Sprague Dawley rats	Theta burst stimulation and paired low-frequency stimulation for LTP, and theta burst stimulation for LTD	Distinct functions of NR2B for LTP and NR2A for LTD seperately.	[19]
Hippocampal, Wistar rats	High-frequency stimulation for LTP and low-frequency stimulation for LTD	Cross-relation of subunits NR2A and NR2B for synaptic plasticity forms LTP and LTD.	[40]
**In vivo** Hippocampus, Sprague Dawley rats	High-frequency stimulation for LTP	Proof for the involvement of the NR2B subunit in LTP.	[41]
Hippocampus, Sprague Dawley rats	High-frequency and theta-burst stimulation for LTP, and low-frequency and paired-burst stimulation for LTD	This study proved the importance of both subunits, NR2A and NR2B, in any form LTP or LTD.	[42]

**Table 2:** The theta burst stimulation (TBS) protocol for induction of LTP in the SC-CA1 synapse: the protocol is composed of long train of 5 bursts of stimulations with 4 pulses at 100 Hz, repeated 5 times, with a pulse train interval of 200 µs.

	Baseline Configuration (−60–0 min)	TBS (at 0 min)	Post-Tetanization Configuration (0–120 min)
**Pulse source**	Voltage	Voltage	Voltage
**# pulse trains**	24×	4×	49×
**pulse train interval**	150 s	20 s	150 s
**pulse length**	200 µs	200 µs	200 µs
**pulse current/voltage**	Variable µA/mV (based on 50% IO curve)	Variable µA/mV (based on 50% IO curve)	Variable µA/mV (based on 50% IO curve)
**burst trains frequency**	/	5 Hz	/
**# burst trains**	/	5×	/
**Burst frequency**	/	100 Hz	/
**# burst pulses**	/	5×	/
**pulse frequency**	0.033 Hz	/	0.033 Hz
**# pulses**	5×	/	5×
**post delay**	150 s	10 s	150 s

## Data Availability

The data that support the findings of this study are available upon reasonable request.
